# Does maintaining apical patency reduce early postoperative pain after root canal treatment? A randomized controlled trial in asymptomatic vital single-rooted teeth

**DOI:** 10.1007/s00784-026-06788-w

**Published:** 2026-02-26

**Authors:** Ozan Arda Deger, Sehnaz Yilmaz, Kübra Gürler

**Affiliations:** Faculty of Dentistry, Department of Endodontics, Cukurova University, Adana, 01330 Adana Turkey

**Keywords:** Apical patency, Endodontics, Postoperative pain, Randomized controlled trial, Root canal treatment

## Abstract

**Objectives:**

The aim of this randomized controlled clinical trial was to evaluate the effects of implementing apical patency (AP) on early postoperative pain in asymptomatic vital single-rooted teeth.

**Materials and methods:**

Seventy-two patients with asymptomatic, vital, single-rooted teeth were randomly assigned to either the patency group or the non-patency group. In the patency group, a #10 K-file was gently extended 1 mm beyond the working length at each instrument change to maintain AP, whereas in the non-patency group, instrumentation was confined within the working length. All treatments were completed at one visit by a single operator using the One Curve NiTi rotary system (25/0.06 or 35/0.04; MicroMega, Besançon, France) and standardized irrigation (2.5% NaOCl, 17% EDTA). Pain was recorded on a Numerical Rating Scale (NRS) at 0–6, 6–12, 12–24, 24–36, and 36–48 h. The data were analyzed using Mann–Whitney U, χ²/Fisher, Friedman, and Generalized Estimating Equations (GEE) tests (α = 0.05).

**Results:**

The groups did not differ significantly in age, gender, or jaw location (*p* > .05). The pain scores were similar at all time intervals (*p* > .05) and decreased significantly over time in both groups (*p* < .001). From 12 to 24 h onward, most patients reported minimal or no pain. Analgesic intake within 48 h was infrequent and comparable between groups (*p* > .05).

**Conclusions:**

In asymptomatic vital single-rooted teeth, maintaining AP did not significantly affect postoperative pain within the first 48 h after single-visit root canal treatment. Therefore, routine use of AP solely to reduce early postoperative pain is not warranted in this patient group.

**Clinical relevance:**

For asymptomatic vital single-rooted teeth, AP should not be performed with the expectation of reducing early postoperative pain; its use should be considered based on case-specific clinical considerations rather than pain control alone.

## Introduction

Postoperative pain following root canal treatment is among the most common complications of endodontic therapy and is often perceived as synonymous with treatment failure, thereby adversely affecting patient satisfaction [1]. The reported prevalence of postoperative pain in the literature ranges from 3% to 58%, a variability attributable to differences in study design, pulpal and periapical status, treatment protocols, instrumentation techniques, and pain assessment methods [2]. Postoperative pain typically peaks within the first 24 h after treatment and subsequently declines over the next 48–72 h [3, 4].

The etiology and pathogenesis of postoperative pain are multifactorial. Mechanical factors (over-instrumentation, apical extrusion), the extrusion of chemical irritants or filling materials, and the translocation of the microbial load into periapical tissues are the principal triggers of the inflammatory response [5, 6]. Apical debris extrusion during chemomechanical preparation has been identified as one of the most critical determinants of periapical inflammation and pain [6]. Accordingly, techniques that reduce debris extrusion and microbial burden within the apical third are of paramount importance in endodontic practice.

Apical patency (AP) is based on the principle of preventing apical blockage and maintaining canal patency by passively passing a small file (#10 K-file) through the apical foramen [7]. First described by Buchanan in 1987, this technique aims to reliably preserve the working length, prevent debris accumulation in the apical region, and facilitate the delivery of irrigants to the apical terminus [8]. It has also been reported to help prevent procedural errors such as ledge formation and canal transportation by preserving the anatomy of the apical constriction while improving the clinician’s tactile sensation [9, 10].

Nevertheless, the clinical application of AP remains contentious [11]. Some investigators argue that the repeated passage of small files beyond the apex may cause mechanical irritation and the extrusion of infected debris, thereby increasing postoperative pain and the incidence of flare-ups [12]. Consequently, some clinicians avoid employing this technique routinely. However, recent clinical studies and systematic reviews have shown that AP does not significantly increase the incidence of postoperative pain and may even contribute to healing by enhancing the efficacy of irrigation [13].

In the current literature, findings regarding the effect of AP on postoperative pain are conflicting [14, 15]. While some randomized controlled trials have reported no significant association between patency and pain, other investigations have suggested a reduction in pain intensity during the early postoperative period [16]. Meta-analyses indicate that definitive conclusions are precluded by methodological heterogeneity; however, they support the view that patency does not exert a clinically meaningful adverse effect on pain [17]. Accordingly, the impact of AP on postoperative pain warrants evaluation through high-quality, well-designed randomized controlled clinical trials. Such studies would clarify whether incorporating this technique into routine endodontic protocols confers genuine clinical benefit, thereby informing evidence-based decision-making.

Given that this trial was restricted to asymptomatic vital teeth without periapical pathology, the baseline microbial/inflammatory burden was expected to be low. Accordingly, the present study primarily addresses whether maintaining apical patency influences early postoperative pain in a low-risk clinical scenario. The aim of this randomized controlled clinical trial was to evaluate the effects of implementing AP on early postoperative pain in asymptomatic vital single-rooted teeth. The null hypothesis of this study is that there is no statistically significant difference in postoperative pain between root canal treatments performed with and without patency.

## Materials and methods

This randomized controlled clinical trial was conducted at the Department of Endodontics, Faculty of Dentistry, Cukurova University, in accordance with the Declaration of Helsinki (and its later amendments). Ethical approval was obtained from the Clinical Research Ethics Committee of the Cukurova University Faculty of Medicine (meeting no.: 144; decision no.: 33; date: May 10, 2024). The study was supported by the Cukurova University Scientific Research Projects Coordination Unit (Project No. TDH-2024-17005) and was registered at ClinicalTrials.gov (NCT07233590). All participants were informed about the study objectives and procedures, and written informed consent was obtained prior to enrollment.

### Sample size calculation

The sample size was calculated using power analysis (G*Power, version 3.1.9.2; Franz Faul, Universität Kiel, Germany), assuming 90% power, a two-sided α of 0.05, and an effect size of 0.8 based on a previous study [18]. The minimum required sample size was determined to be 30 patients per group. In anticipation of potential dropouts in the postoperative period, the sample size was increased by 20%, and a total of 72 patients were planned for inclusion and randomly allocated into two groups (*n* = 36).

## Inclusion criteria

- Individuals aged 18–65 years, classified as ASA I or ASA II according to the American Society of Anesthesiologists (ASA), who had not taken analgesics for at least 6 h prior to treatment.
- Asymptomatic, single-rooted vital teeth without spontaneous pain, including teeth with iatrogenic pulp exposure during prosthetic tooth preparation (e.g., crown preparation) or teeth adjacent to a cyst in an area scheduled for cyst surgery.
- Radiographic evidence of mature apices with no periapical pathology; periodontal probing depth ≤ 3 mm; and gingival health meeting Glickman’s criteria [19].
- Individuals who are literate, able to provide written informed consent, and capable of understanding and using the pain scale.

## Exclusion criteria

- Patients who are pregnant or have a cardiac pacemaker.
- Patients who used antibiotics within the past month or who required antibiotic prophylaxis.
- Presence of extensive restorations in the tooth of interest.
- Patients reporting concurrent pain in adjacent teeth.
- Dental or orofacial pain of non-endodontic origin.

## Group randomization

Randomization was performed using an online software tool with block randomization (block size of ten) to ensure equal allocation between groups (www.randomizer.org).

Patients were randomly assigned to two groups:

1. Patency group.
2. Non-patency group.

## Clinical procedure

Preoperative pain levels were recorded using a Numerical Rating Scale (NRS; 0–10, where 0 = no pain and 10 = worst imaginable pain) prior to endodontic procedures. All anesthetic injections and dental procedures were performed during a single visit by a single operator (O.A.D.) under magnification (3× loupes) and illumination. Anesthesia was achieved with 4% articaine hydrochloride containing 1:100,000 epinephrine (Ultracain D-S; Sanofi, Paris, France). Buccal infiltration anesthesia was administered for single-rooted maxillary teeth and mandibular anterior teeth, whereas an inferior alveolar nerve block was used for mandibular premolars. Following successful anesthesia, the tooth was isolated with a rubber dam. Any caries or coronal restorations were completely removed. An endodontic access cavity was prepared under copious water cooling using high-speed sterile diamond burs (#801G, Meisinger).

All subsequent endodontic procedures from access cavity preparation to root canal obturation were performed in the following groups:

1. Patency group (*n* = 36): The #10 K-file used for the measurements was carefully advanced into the canal until the electronic apex locator indicated the apex (red zone). Once “APEX” appeared on the display, the file was slowly withdrawn until the Root ZX Mini (Root ZX Mini, Morita, Tokyo, Japan) screen showed “00” in green, at which point advancement ceased. The measurement was considered accurate when the instrument was held steady for at least 5 s and the “00” reading remained unchanged. The file position was stabilized with a silicone stop and measured with an endodontic ruler, and the working length (WL) was established and then verified with a periapical radiograph. In teeth where a clear indication of the apical constriction could not be obtained, the measurement was repeated using a #15 or #20 K-file. Following this step in the patency group, a #10 K-file was gently extended 1 mm beyond the WL to maintain AP, remove accumulated debris, and facilitate irrigant delivery to the apical terminus. This patency maneuver was repeated at each instrument change throughout canal shaping.
2. Non-patency group (*n* = 36): The #10 K-file used for measurement was carefully advanced into the canal until the electronic apex locator signaled the apex (red zone). Once “APEX” appeared on the display, the file was slowly withdrawn until “00” in green was observed on the Root ZX Mini (Root ZX Mini, Morita, Tokyo, Japan), at which point advancement was terminated. The measurement was considered accurate when the instrument was held steady for at least 5 s and the “00” reading remained unchanged. The file position was stabilized with a silicone stop and measured with an endodontic ruler, and the working length (WL) was established and then verified with a periapical radiograph. In teeth where a definite “tug-back” sensation at the apical constriction could not be achieved, the measurement was repeated using a #15 or #20 K-file.

After the working length was determined, all canals were shaped using the One Curve NiTi rotary system (25/0.06 or 35/0.04; MicroMega, Besançon, France) with a crown-down technique. Considering root canal length and apical foramen size, the apical preparation was standardized to size #25 or #35, and rotary files of 25–31 mm length were selected accordingly. The instruments were operated at low speed and torque (300 rpm, 2 N·cm) and removed for cleaning after every three pecking motions. Following each shaping step, canals were irrigated with 2 mL of 2.5% sodium hypochlorite (NaOCl) (Wizard; Rehber Kimya) using 30-gauge side-vented needles (NaviTips; Ultradent). These procedures were repeated until the instruments reached the working length. A total of 10 mL of 2.5% NaOCl was used per canal during shaping.

The final rinse was performed sequentially with 2 mL of 2.5% NaOCl, 2 mL of 17% ethylenediaminetetraacetic acid (EDTA) (Endo-Solution; PPH Cerkamed), and 2 mL of distilled water. After the canals were dried with paper points (President Dental), root canal obturation was completed using cold lateral compaction with gutta-percha (President Dental) and an epoxy resin–based sealer (AH Plus; Dentsply Maillefer, Ballaigues, Switzerland). Definitive restorations were placed using composite resin (GC SolareX; GC Corporation, Tokyo, Japan). The quality of obturation was verified with a periapical radiograph. Each patient was prescribed 400 mg of ibuprofen to be taken as needed and was instructed to return to the clinic if severe postoperative pain persisted despite analgesic use.

### Postoperative evaluation

Postoperative pain assessment was performed by a blinded investigator (S.Y.). All participants were given a questionnaire to record their pain on the NRS at 0–6, 6–12, 12–24, 24–36, and 36–48 h, as well as to document any analgesic intake. Patients were recalled 48 h after treatment for clinical evaluation and collection of the completed NRS forms.

### Statistical analysis

All statistical analyses were performed using SPSS software version 20.0 (IBM Corp., Armonk, NY, USA). The distribution of continuous variables was assessed with the Shapiro–Wilk test. For non-normally distributed data, intergroup comparisons were conducted using the Mann–Whitney U test. Categorical variables, including gender and analgesic intake, were compared using the Pearson Chi-square test or Fisher’s exact test, as appropriate. Changes in NRS scores over time were analyzed with the Friedman test, and repeated measures were further evaluated using Generalized Estimating Equations (GEE) models. The level of statistical significance was set at *p* < .05.

## Results

**Fig. 1:**
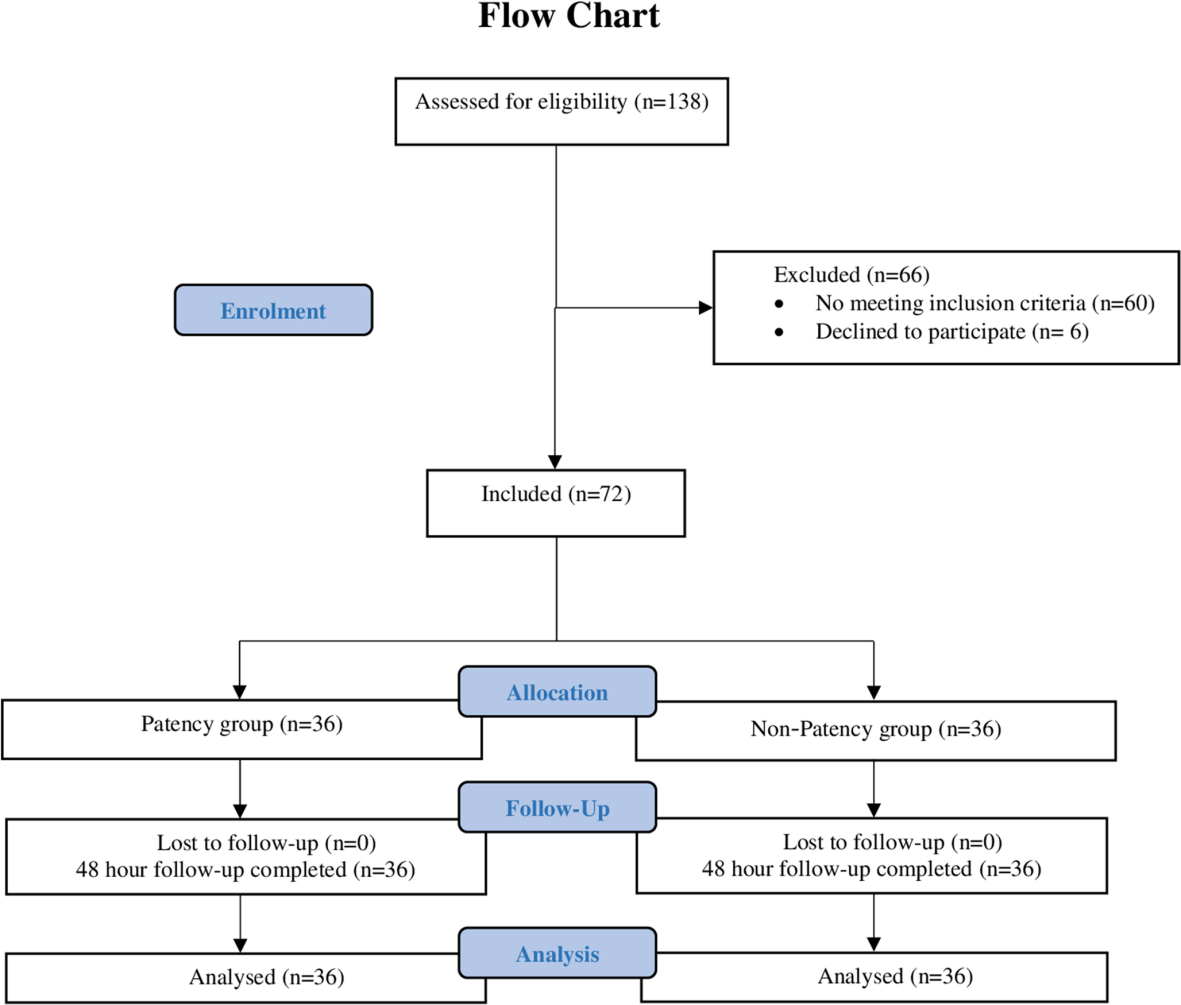
CONSORT flow diagram of participant enrollment, allocation, follow-up, and analysis

A total of 72 patients aged 18–65 years who met the inclusion criteria were enrolled (37 females, 35 males). Among these patients, 36 were assigned to the patency group, and 36 were assigned to the non-patency group **(**Fig. 1**)**. The groups were comparable with respect to age, gender distribution, and jaw location, with no statistically significant differences between them (*p* > .05) **(**Table 1**)**.

**Table 1:** Distribution of patients by gender, age, and jaw (arch) between the patency and non-patency groups. Categorical variables are presented as n (%), and age is presented as mean ± SD and median (min–max)

Study	Patency	Non-patency	*p* Value
Groups	(*n* = 36)	(*n* = 36)	
Gender, n (%)			
Female	22 (61)	18 (50)	.343^a^
Male	14 (39)	18 (50)	
Age			
(Mean ± SD)	42.3 ± 14.0	39.6 ± 13.1	.406^b^
Median (min-max)	45 (30–55)	40 (28.5–50)	
Jaw (arch), n (%)			
Maxilla	14 (39)	23 (64)	0.034^a^
Mandible	22 (61)	13 (36)	

^a^ χ2 Test

^b^ Mann-Whitney U Test

The mean preoperative NRS scores were higher in the patency group than in the non-patency group; however, this difference was not statistically significant (*p* > .05). During the 0–6-hour interval, the mean NRS scores were lower in the non-patency group than in the patency group, but the difference was not statistically significant (*p* > .05). The pain scores decreased markedly over time in both groups. From 12 to 24 h, the vast majority of patients reported minimal pain or no pain (NRS = 0), and nearly all patients were pain free after 24 h **(**Table 2**)**.

**Table 2:** Comparison of NRS pain scores between the patency and non-patency groups at baseline (preoperative) and across postoperative time intervals (0–6, 6–12, 12–24, 24–36, and 36–48 h). Data are presented as mean ± SD and median (min–max)

	Patency	Non-patency	*p* Value^δ^
	mean ± SD / median (min-max)	mean ± SD / median (min-max)	
Pre-op Pain	1.75 ± 1.27 / 2 (0.5-3)^Aa^	1.17 ± 1.25 / 1 (0-2.5)^Aa^	0.052
0–6 h	1.64 ± 1.17 / 1.5 (1–3)^Aa^	1.17 ± 1.21 / 1 (0–2)^Aa^	0.084
6–12 h	0.92 ± 1.00 / 1 (0–2)^Aac^	0.69 ± 1.01 / 0 (0–1)^Aac^	0.242
12–24 h	0.34 ± 0.54 / 0 (0–1)^Abc^	0.39 ± 0.84 / 0 (0–0)^Abc^	0.562
24–36 h	0.17 ± 0.45 / 0 (0–0)^Ab^	0.14 ± 0.42 / 0 (0–0)^Abc^	0.731
36–48 h	0.08 ± 0.37 / 0 (0–0)^Abc^	0.06 ± 0.23 / 0 (0–0)^Abc^	0.977
*p* Value^φ^	< 0,001	< 0,001	

^δ^ Mann Whitney U test, ^φ^ Friedman Test

A: For between-group comparisons at the same time point (within each row), values with different uppercase letters indicate a statistically significant difference (*p* < .05), whereas values sharing the same uppercase letter indicate no significant difference, a-c: For within-group comparisons across time points (within each column), values that do not share any lowercase letter are significantly different (*p* < .05). Values that share at least one lowercase letter are not significantly different.

The Friedman test indicated a significant reduction in pain scores over time in both groups (*p* < .001). However, Generalized Estimating Equations (GEE) analysis revealed no significant difference in the rate of pain reduction between the groups *(p* > .05) **(**Table 3**)**. 

**Table 3:** Time-interval distribution of NRS pain scores (0–4) in the patency and non-patency groups, presented as n (%), for the preoperative period and postoperative intervals (0–6, 6–12, 12–24, 24–36, and 36–48 h). Between-group differences at each interval were evaluated using the Pearson χ² test.Within-group changes over time were analyzed using Generalized Estimating Equations (GEE), with corresponding p values reported for each group

	Patency	Non-patency	*p*Value^ϴ^
Pre-op Pain			
0	9 (23)	16 (44)	.209
1	9 (23)	7 (19)	
2	4 (10)	4 (11)	
3	17 (44)	9 (25)	
0-6 hours			
0	8 (21)	15 (42)	.312
1	13 (33)	8 (22)	
2	7 (18)	5 (14)	
3	10 (26)	8 (22)	
4	1 (3)	0 (0)	
6-12 hours			
0	18 (46)	22 (61)	.577
1	11 (28)	6 (17)	
2	7 (18)	5 (14)	
3	3 (8)	3 (8)	
12-24 hours			
0	27 (71)	28 (78)	.197
1	10 (26)	4 (11)	
2	1 (3)	2 (6)	
3	0 (0)	2 (6)	
24-36 hours			
0	34 (87)	32 (89)	.999
1	4 (10)	3 (8)	
2	1 (3)	1 (3)	
36-48 hours			
0	37 (95)	34 (94)	.797
1	1 (3)	2 (6)	
2	1 (3)	0 (0)	
*p*Value^Ʃ^	.045	.005	

^ϴ^Pearson χ2 Test,^Ʃ^*GEE* Generalized estimating equations

Within the first 48 postoperative hours, only four patients (two in each group) required analgesics. There was no statistically significant difference in analgesic intake between the study groups (*p* > .05) **(**Table 4**)**.

**Table 4:** Distribution of the number of analgesic tablets taken within 0–48 h after treatment (0, 1, and 2 tablets) in the patency and non-patency groups, presented as n (%)

	Patency	Non-patency	Total	*p* Value
	(*n* = 36)	(*n* = 36)	(*n* = 72)	
Analgesic tablets taken, n (%)				
0	34 (94)	34 (94)	68 (94)	1.000^*^
1	1 (3)	1 (3)	2 (3)	
2	1 (3)	1 (3)	2 (3)	

* Fisher’s exact test

## Discussion

During root canal instrumentation, AP is maintained with a patency file to prevent debris accumulation and potential blockage in the apical region [17]. However, evidence regarding the effect of AP on post-endodontic pain is inconsistent, and its impact remains uncertain. This randomized controlled clinical trial evaluated the effect of maintaining AP on postoperative pain in asymptomatic vital single-rooted teeth. The principal finding was that AP had no clinically meaningful effect on pain intensity or analgesic consumption during the first 48 h. In both groups, the pain scores decreased significantly over time, and the vast majority of the patients were pain free from 24 h onward. Although NRS scores appeared lower in the non-patency group during the earliest 0–6-hour interval, this difference was not statistically significant; during the subsequent 6–24 h, pain declined markedly in both groups, and the between-group difference disappeared. On the basis of these findings, the null hypothesis that “there is no difference in postoperative pain between treatments with patency and non-patency” was statistically accepted.

Because the present trial was restricted to asymptomatic vital teeth without periapical pathology, bacterial infection and periapical inflammation were likely minimal at baseline. Under such conditions, maintaining apical patency may be less likely to trigger additional inflammatory responses that could translate into postoperative pain. This population characteristic may partially explain the absence of intergroup differences. Additionally, both groups were shaped to the established working length using the same rotary instrumentation and irrigation protocol, which may have minimized procedural variability and attenuated any incremental effect of apical patency on pain outcomes.

The size of the file used in the patency protocol is a pivotal variable. The report by Cailleteau and Mullaney on the teaching of patency in U.S. dental schools revealed inter-institutional variation in the instrument size used to maintain apical foramen patency: 42% of programs use a #10, 33% a #15, and 25% a #20 K-file. In the present study, AP was routinely maintained with a #10 K-file. Choosing larger sizes may traumatize periapical tissues, complicate control of the obturation technique, and increase the extrusion of infected debris, thereby increasing the incidence of post-endodontic pain and adversely affecting treatment outcomes. Indeed, Goldberg and Masson reported that when a #20 K-file was used as the patency instrument, the probability of apical foramen transportation reached 56.6% [20].

Potential risk factors associated with postoperative pain flare-ups include patient age and gender, pulpal status, type and concentration of irrigant used, tooth type, number of treatment visits, and instrumentation approach [21]. These variables may constitute major sources of between-study heterogeneity. The age range of patients included in the present study (18–65 years) was relatively broad. Findings in the literature regarding age and sex are inconsistent: one study reported the highest incidence of postoperative pain in the 18–33-year range [22], whereas others reported peak incidence in the 50–60-year group [23, 24]; another study reported the greatest risk in patients aged 41–65 years [25]. Conversely, some studies have reported no associations between pain incidence and either age or gender [26]. In addition, several investigations suggest that women may be more prone to flare-ups than men are, potentially due to a lower pain threshold [25, 27].

Pulpal status has also emerged as a potential determinant of postoperative pain incidence. Some studies have reported a positive association between the presence of necrotic pulp and postoperative pain [23, 26, 28], whereas others have reported no significant relationship between pulpal status and pain [25, 29, 30]. These conflicting findings suggest that pulpal status alone may not be decisive in the pathogenesis of pain and that additional patient- and treatment-specific factors are likely involved. In the present study, the sample consisted of asymptomatic, single-rooted vital teeth without spontaneous pain; therefore, the findings should be interpreted within this clinical context.

Variations in the type and scope of irrigation protocols can also produce meaningful variability in postoperative pain outcomes. A recent systematic review and meta-analysis reported a postoperative pain incidence of 45% in patients irrigated with low-concentration (0.5–3%) NaOCl, compared with 39% at higher concentrations (5–8.25%) [31]. Conversely, some studies have reported no significant associations between postoperative pain and either differing NaOCl concentrations or alternative irrigants [32, 33]. Numerous investigations have additionally examined irrigant penetration into the apical third. Vera et al. radiographically demonstrated that maintaining AP in wide canals increases irrigant penetration into the apical 2 mm of the root [10]. Similarly, Vera et al. and Kamra et al. reported that preserving AP, particularly when combined with passive ultrasonic irrigation, enhances delivery of the irrigant to the apical third; this may improve canal cleanliness and thereby contribute to reduced postoperative pain [10, 34]. In the present study, the irrigation regimen was systematically standardized across groups. This approach aimed to minimize the confounding effects introduced by the protocol heterogeneity reported in the literature and to isolate AP status as the sole independent variable in the analysis. Consistent use of a medium-strength (2.5%) NaOCl solution and controlled root canal irrigation with side-vented needles mitigated the risk of irrigant extrusion, thereby balancing potential irrigation-related effects on postoperative pain.

The anatomical position of the tooth, functional loading, and nature of occlusal forces may influence pain tolerance after endodontic treatment [35]. The literature indicates that tooth type can be a determinant of postoperative pain due to increased canal complexity and a greater number of canals [5, 36]. Mandibular molars, in particular, carry a greater risk of pain because of the relatively thicker cortical bone [37]; this anatomical feature may restrict exudate drainage and reduce regional blood flow, thereby delaying healing [25]. In contrast, in the present study, the selection of exclusively vital single-rooted teeth limited anatomical and microbial variability, allowing the protocol’s dominant effect on pain dynamics to emerge.

There is evidence in the literature that the number of treatment visits may influence the incidence of post-endodontic pain [26, 38]. In single-visit root canal treatments where AP was maintained, no significant differences in postoperative pain scores were reported compared with those in non-patency groups [39, 40]; however, Yaylalı et al. reported higher mean pain scores in the non-patency group [15]. Findings in multi-visit treatments are variable: Sharaan et al. reported greater pain at 6 and 12 h in the patency group [41], whereas Arora et al. reported less pain in the patency group on days 1, 2, 3, and 4 [18]. In the present study, all root canal treatments were performed at a single visit, and no statistically significant difference in postoperative pain scores was detected between the patency and non-patency groups. Because all the patients were treated in a single session, the number of visits did not vary and was therefore not included as an independent variable in the multivariable analyses.

Debris extrusion generated during root canal preparation depends on the instruments and technology employed; lower debris levels are generally associated with reduced postoperative pain [42]. Compared with hand instruments, rotary systems typically result in less debris extrusion; consequently, the incidence and severity of postoperative pain are lower with rotary instrumentation [43]. In the present study, canals in both groups were shaped using the One Curve NiTi rotary system according to a crown-down approach, and a standardized irrigation protocol with 2.5% NaOCl and 17% EDTA was applied. This modern, controlled biomechanical protocol may reduce the microbial and mechanical load in the apical region to low levels, thereby attenuating any additional effect of AP and helping to explain the absence of differences between groups.

This study has several limitations. First, because pain monitoring was limited to 0–48 h, potential between-group differences at later time points (3–7 days) and possible late flare-up events could not be detected. Although restricting the sample to vital, asymptomatic, single-rooted teeth enhances internal validity, it limits the generalizability of the findings to teeth with necrotic pulps, symptomatic presentations, or multiple roots. Future trials including necrotic/infected teeth and longer follow-up periods are warranted to determine whether the findings extend to higher-risk clinical scenarios. Despite the advantages of single-center, single-operator standardization, external validity remains limited due to operator effects and inter-institutional variations in clinical practice. The irrigation protocol was standardized in all cases using 2.5% NaOCl and 17% EDTA delivered with a 30G side-vented needle; because different concentrations, activation methods, or needle types were not compared, inferences regarding irrigation parameters are constrained. Additionally, procedural covariates such as sealer extrusion, master apical file size, and primary apical diameter were not prospectively collected for adjusted analyses and should be prioritized in future trials. Finally, pain was assessed by patient self-reports using the NRS, an approach susceptible to perceptual biases and not corroborated by biomarkers or radiographic outcomes.

## Conclusion

In single-visit treatment of asymptomatic vital, single-rooted teeth performed with standard NiTi rotary instrumentation and an effective irrigation regimen, routine implementation of AP solely to reduce early postoperative pain within the first 48 h does not appear warranted. Accordingly, AP should be employed selectively on the basis of patient- and tooth-specific risk–benefit assessments.

## Data Availability

The datasets generated and/or analysed during the current study are not publicly available due to ethical restrictions and patient confidentiality but are available from the corresponding author on reasonable request.

## References

[1] Rosenberg PA (2002) Clinical strategies for managing endodontic pain. Endodontic Top 3:78–92

[2] Sathorn C, Parashos P, Messer H (2008) The prevalence of postoperative pain and flare-up in single‐and multiple‐visit endodontic treatment: a systematic review. Int Endod J 41:91–99 17956561 10.1111/j.1365-2591.2007.01316.x

[3] Oliveira PS, Ferreira MC, Paula NGN et al (2022) Postoperative pain following root canal instrumentation using protaper next or reciproc in asymptomatic molars: a randomized controlled single-blind clinical trial. J Clin Med 11:3816 35807101 10.3390/jcm11133816 PMC9267392

[4] Machado R, Comparin D, Ignácio SA, da Silva Neto UX (2021) Postoperative pain after endodontic treatment of necrotic teeth with large intentional foraminal enlargement. Restor Dent Endod 46(3):e31 34513637 10.5395/rde.2021.46.e31 PMC8411006

[5] Torabinejad M, Kettering JD, McGraw JC, Cummings RR, Dwyer TG, Tobias TS (1988) Factors associated with endodontic interappointment emergencies of teeth with necrotic pulps. J Endod 14:261–266 3251982 10.1016/S0099-2399(88)80181-X

[6] Seltzer S, Naidorf IJ (1985) Flare-ups in endodontics: I. Etiological factors. J Endod 11:472–478 3868692 10.1016/S0099-2399(85)80220-X

[7] American Association of Endodontists (2003) Glossary of endodontic terms, 7th edn. American Association of Endodontists, Chicago, IL

[8] Buchanan LS (1987) Working length and apical patency: the control factors. Endod Rep Fall-Winter 16–20 3481573

[9] Buchanan LS (1989) Management of the curved root canal. J Calif Dent Assoc 17(4):18–25 2481012

[10] Vera J, Hernández EM, Romero M, Arias A, van der Sluis LW (2012) Effect of maintaining apical patency on irrigant penetration into the apical two millimeters of large root canals: an in vivo study. J Endod 38(10):1340–1343 22980174 10.1016/j.joen.2012.06.005

[11] Mohammadi Z, Jafarzadeh H, Shalavi S, Kinoshita JI (2017) Establishing apical patency: to be or not to be? J Contemp Dent Pract 18(4):326–329 28349913 10.5005/jp-journals-10024-2040

[12] Cailleteau JG, Mullaney TP (1997) Prevalence of teaching apical patency and various instrumentation and obturation techniques in united States dental schools. J Endod 23:394–396 9545951 10.1016/S0099-2399(97)80191-4

[13] Xiqian L, Ying Z, Mian M (2024) The effect of apical patency on postoperative pain following endodontic therapy: a systematic review and meta-analysis. Eur J Oral Sci 132:e12986 38632110 10.1111/eos.12986

[14] Shubham S, Nepal M, Mishra R, Dutta K (2021) Influence of maintaining apical patency in post-endodontic pain. BMC Oral Health 21(1):284 34078331 10.1186/s12903-021-01632-x PMC8173919

[15] Yaylali IE, Kurnaz S, Tunca YM (2018) Maintaining apical patency does not increase postoperative pain in molars with necrotic pulp and apical periodontitis: a randomized controlled trial. J Endod 44:335–340 29370942 10.1016/j.joen.2017.11.013

[16] Yousaf A, Ali F, Bhangar F, Alam M (2021) Effect of apical patency on postoperative pain after Single-visit endodontic treatment in necrotic teeth with asymptomatic apical periodontitis: A randomised control trial. J Coll Physicians Surg Pak 31(10):1154–1158 34601833 10.29271/jcpsp.2021.10.1154

[17] Abdulrab S, Rodrigues JC, Al-Maweri SA, Halboub E, Alqutaibi AY, Alhadainy H (2018) Effect of apical patency on postoperative pain: A Meta-analysis. J Endod 44(10):1467–1473 30170845 10.1016/j.joen.2018.07.011

[18] Arora M, Sangwan P, Tewari S, Duhan J (2016) Effect of maintaining apical patency on endodontic pain in posterior teeth with pulp necrosis and apical periodontitis: a randomized controlled trial. Int Endod J 49:317–324 25866134 10.1111/iej.12457

[19] Glickman I (1972) Clinical periodontology: prevention, diagnosis, and treatment of periodontal disease in the practice of general dentistry, 4th edn. Saunders, Philadelphia

[20] Goldberg F, Massone EJ (2002) Patency file and apical transportation: an in vitro study. J Endod 28(7):510–511 12126377 10.1097/00004770-200207000-00005

[21] Bassam S, El-Ahmar R, Salloum S, Ayoub S (2021) Endodontic postoperative flare-up: an update. Saudi Dent J 33(7):386–394 34803278 10.1016/j.sdentj.2021.05.005 PMC8589595

[22] Naoum HJ, Chandler NP (2002) Temporization for endodontics. Int Endod J 35(12):964–978 12653314 10.1046/j.1365-2591.2002.00600.x

[23] Azim AA, Azim KA, Abbott PV (2017) Prevalence of inter-appointment endodontic flare-ups and host-related factors. Clin Oral Investig 21(3):889–894 27179654 10.1007/s00784-016-1839-7

[24] Nair M, Rahul J, Devadathan A, Mathew J (2017) Incidence of endodontic Flare-ups and its related factors: A retrospective study. J Int Soc Prev Community Dent 7(4):175–179 28852632 10.4103/jispcd.JISPCD_61_17 PMC5558250

[25] Ali SG, Mulay S, Palekar A, Sejpal D, Joshi A, Gufran H (2012) Prevalence of and factors affecting post-obturation pain following single visit root canal treatment in Indian population: A prospective, randomized clinical trial. Contemp Clin Dent 3(4):459–463 23633809 10.4103/0976-237X.107440 PMC3636834

[26] Onay EO, Ungor M, Yazici AC (2015) The evaluation of endodontic flare-ups and their relationship to various risk factors. BMC Oral Health 15(1):142 26577095 10.1186/s12903-015-0135-2 PMC4647657

[27] Garcia-Font M, Durán-Sindreu F, Morelló S, Irazusta S, Abella F, Roig M, Olivieri JG (2018) Postoperative pain after removal of gutta-percha from root canals in endodontic retreatment using rotary or reciprocating instruments: a prospective clinical study. Clin Oral Investig 22(7):2623–2631 29396645 10.1007/s00784-018-2361-x

[28] Gotler M, Bar-Gil B, Ashkenazi M (2012) Postoperative pain after root canal treatment: a prospective cohort study. Int J Dent 2012:310467 10.1155/2012/310467 PMC3312224 22505897

[29] Sevekar SA, Gowda SHN (2017) Postoperative pain and Flare-Ups: comparison of incidence between single and multiple visit pulpectomy in primary molars. J Clin Diagn Res 11(3):ZC09–ZC12 28511499 10.7860/JCDR/2017/22662.9377 PMC5427425

[30] Pasqualini D, Mollo L, Scotti N, Cantatore G, Castellucci A, Migliaretti G, Berutti E (2012) Postoperative pain after manual and mechanical glide path: a randomized clinical trial. J Endod 38(1):32–36 22152616 10.1016/j.joen.2011.09.017

[31] Sabino-Silva R, Cardoso IV, Vitali FC, Alves AMH, Souza BDM, Bortoluzzi EA, da Fonseca Roberti Garcia L, da Silveira Teixeira C (2023) Prevalence of postoperative pain after endodontic treatment using low and high concentrations of sodium hypochlorite: a systematic review and meta-analysis. Clin Oral Investig 27(8):4157–4171 37466716 10.1007/s00784-023-05151-7

[32] Demenech LS, de Freitas JV, Tomazinho FSF, Baratto-Filho F, Gabardo MCL (2021) Postoperative pain after endodontic treatment under irrigation with 8.25% sodium hypochlorite and other solutions: A randomized clinical trial. J Endod 47(5):696–704 33607121 10.1016/j.joen.2021.02.004

[33] Verma N, Sangwan P, Tewari S, Duhan J (2019) Effect of different concentrations of sodium hypochlorite on outcome of primary root canal treatment: A randomized controlled trial. J Endod 45(4):357–363 30827769 10.1016/j.joen.2019.01.003

[34] Kamra AI, Tank JM, Banga KS (2016) Effect of maintaining apical patency and passive ultrasonic irrigation on irrigant penetration into the apical third of root canals: an in vivo study. Endodontology 28(2):127–131

[35] Bates JF, Stafford GD, Harrison A (1976) Masticatory function - a review of the literature. III. Masticatory performance and efficiency. J Oral Rehabil 3(1):57–67 772184 10.1111/j.1365-2842.1976.tb00929.x

[36] Ng YL, Glennon JP, Setchell DJ, Gulabivala K (2004) Prevalence of and factors affecting post-obturation pain in patients undergoing root canal treatment. Int Endod J 37(6):381–391 15186245 10.1111/j.1365-2591.2004.00820.x

[37] Alí A, Olivieri JG, Duran-Sindreu F, Abella F, Roig M, García-Font M (2016) Influence of preoperative pain intensity on postoperative pain after root canal treatment: A prospective clinical study. J Dent 45:39–42 26678517 10.1016/j.jdent.2015.12.002

[38] Su Y, Wang C, Ye L (2011) Healing rate and post-obturation pain of single- versus multiple-visit endodontic treatment for infected root canals: a systematic review. J Endod 37(2):125–132 21238790 10.1016/j.joen.2010.09.005

[39] Arias A, Azabal M, Hidalgo JJ, de la Macorra JC (2009) Relationship between postendodontic pain, tooth diagnostic factors, and apical patency. J Endod 35(2):189–192 19166770 10.1016/j.joen.2008.11.014

[40] Garg N, Sharma S, Chhabra A, Dogra A, Bhatia R, Thakur S (2017) Clinical evaluation of maintenance of apical patency in postendodontic pain: an in vivo study. Endodontology 29(2):115–119

[41] Sharaan ME, Aboul-Enein NM (2012) Relationship between post-preparation pain and apical patency: a randomized clinical trial. Gulf Med J 1:S96–101

[42] Tanalp J, Güngör T (2014) Apical extrusion of debris: a literature review of an inherent occurrence during root canal treatment. Int Endod J 47:211–221 23711187 10.1111/iej.12137

[43] Motlani M, Prasad PK, Makkad RS, Nair R, Khiyani S, Batra S (2021) Incidence and severity of postoperative pain following root canal treatment in nonvital pulps with hand and rotary instrumentation techniques in Chhattisgarh population. J Pharm Bioallied Sci 13(Suppl 1):S319–S322 34447102 10.4103/jpbs.JPBS_711_20 PMC8375855

